# Systems Biology and Multi-Omics Determinants of Response to Bladder-Preserving Trimodality Therapy in Muscle-Invasive Bladder Cancer

**DOI:** 10.3390/life16050826

**Published:** 2026-05-16

**Authors:** Vlad-Horia Schițcu, Vlad Cristian Munteanu, Mihnea Bogdan Borz, Ion Cojocaru, Octavia Morari, Mircea Gîrbovan, Andrei-Ionuț Tișe

**Affiliations:** 1 Department of Urology, Institute of Oncology “Prof. Dr. Ion Chiricuta” Cluj-Napoca, 400015 Cluj-Napoca, Romania; 2 Department of Anatomy and Embryology, Iuliu Hatieganu University of Medicine and Pharmacy, 400012 Cluj-Napoca, Romania; 3 Department of Urology, Targu Mures County Clinical Hospital, 540136 Targu Mures, Romania; 4 Department of Anatomy, George Emil Palade University of Medicine, Pharmacy, Science, and Technology of Targu Mures, 540139 Targu Mures, Romania; 5 Department of Urology, Galati County Emergency Hospital, 800578 Galati, Romania; 6 Department of Urology, Alba County Emergency Hospital, 711325 Alba-Iulia, Romania

**Keywords:** bladder preservation, chemoradiation, trimodality therapy, salvage cystectomy, *ERCC2*, DNA damage response, molecular subtypes, immune contexture, radiomics, digital pathology, interpretable machine learning

## Abstract

Trimodality therapy (TMT)—maximal transurethral resection of bladder tumor (TURBT) followed by concurrent chemoradiotherapy—can offer oncologic outcomes comparable to radical cystectomy (RC) in carefully selected muscle-invasive bladder cancer (MIBC) patients while preserving the bladder and, possibly, the quality of life. Systematic reviews and long-term series support durable bladder-intact survival in responders, yet there is still a significant percentage of patients who exhibit incomplete response or invasive intravesical recurrence requiring salvage RC. This review covers computational genomics, transcriptomics, immune contexture, radiogenomics, and digital pathology approaches for predicting response in order to avoid preventable TMT failures. We discuss clinically relevant endpoints (complete response, invasive recurrence, bladder-intact survival, and salvage RC), patient selection (carcinoma in situ, hydronephrosis, debulking feasibility, and histology), and DNA damage response (DDR) biology—highlighting *ERCC2* and related pathways as determinants of chemo-radiation sensitivity. We then review reproducible transcriptomic subtype classifiers and immune deconvolution methods, emphasizing translational constraints and reporting standards. Finally, we propose an integrated hypothetical modeling framework (calibration, external validation, and decision-curve thresholds) to guide recommendations for upfront RC versus bladder preservation with intensified surveillance and timely salvage RC.

## 1. Introduction

Bladder cancer continues to impose a substantial global burden; contemporary estimates of incidence and mortality are summarized in the GLOBOCAN 2020 analysis [1]. Muscle-invasive bladder cancer (MIBC) represents a biologically aggressive disease that even with definitive therapy, the reported 5-year overall survival commonly remains in the range of ~50–60%, whereas in the absence of curative-intent management median survival has been described as approximately 8–11 months. Clinical decision-making in MIBC is thus repeatedly confronted with a familiar dilemma: maximizing cure while limiting treatment-related morbidity and preserving long-term quality of life. Moreover, sex-associated differences in etiology and outcomes add further complexity to risk estimation and survivorship planning [2].

Bladder-preserving trimodality therapy (TMT), which entails maximal transurethral resection followed by concurrent chemoradiation, has been proposed as a curative-intent alternative for selected patients, supported by systematic reviews and mature multi-centric data series [3,4,5]. It is suggested that, in appropriately selected cohorts, long-term oncologic outcomes with TMT may resemble those achieved with radical cystectomy (RC), with or without neoadjuvant chemotherapy; nonetheless, the interpretability of these comparisons is constrained by selection effects, staging heterogeneity, and non-uniform endpoint definitions across studies [3,6]. Decision-analytic models reinforce that even when disease-specific outcomes appear broadly similar, patient preference can shift the effective strategy, underscoring the preference-sensitive nature of organ preservation versus radical treatment [7].

Yet the promise of bladder preservation is tempered by the clinical consequences of failure. Incomplete response to induction, invasive intravesical recurrence, or progression can necessitate delayed definitive surgery and may increase procedural complexity. In this setting, salvage cystectomy is not an exceptional event but a recognized component of TMT pathways, and meta-analytic data provide estimates of its incidence and associated outcomes after bladder-sparing therapy [8].

Despite improvements in imaging, clinical staging—essential for treatment modality selection—remains imprecise, with clinical-to-pathologic stage discordance documented in up to 75% of patients undergoing radical cystectomy (RC). In a large National Cancer Database analysis (*n* = 16,953), 41.9% of patients were pathologically upstaged at RC, with upstaging independently associated with increased 5-year mortality (hazard ratio 1.80; *p* < 0.001) [9]. More recent data presented at EAU 2025 confirmed that among patients who did not receive neoadjuvant therapy, only 25.3% demonstrated concordance between clinical and pathologic stage; 44.8% were upstaged and 29.9% were downstaged at RC [10].

Against this background, the limits of clinicopathological stratification alone—coupled with the rapid expansion of transcriptomic, genomic, and immune profiling—have sharpened interest in computational decision support. It appears increasingly plausible that integrating molecular subtype, DNA damage response (DDR) phenotypes, and immune contexture could refine pre-treatment counseling and identify patients in whom early conversion to cystectomy should be contemplated. The present review appraises the current evidence for genomic and bioinformatic predictors of TMT response and outlines a precision framework for cystectomy timing in MIBC.

## 2. Materials and Methods

This article is a narrative (scoping) review focused on molecular and computational predictors of response to bladder-preserving trimodal therapy in muscle-invasive bladder cancer and their potential integration into decision-making regarding radical cystectomy. We considered English-language studies published from January 2000 to January 2026.

We searched PubMed/MEDLINE, Embase, Web of Science, and the Cochrane Library using combinations of controlled vocabulary and free-text terms related to MIBC and bladder preservation, including: “muscle-invasive bladder cancer”, “MIBC”, “trimodal therapy”, “bladder-preserving chemoradiation”, “MRE11”, “molecular subtype”, “basal luminal”, “consensus classification”, “radiation response”, “immune infiltration”, “multiomics”, and “decision support”. References of key reviews, guideline documents, and seminal clinical trials were manually screened for additional relevant publications.

We prioritized studies that: (i) included MIBC cohorts treated with TMT or chemoradiation where genomic, transcriptomic, or protein biomarkers were linked to treatment outcomes; (ii) investigated MRE11 or other DDR markers as predictors of radiation sensitivity; (iii) evaluated molecular subtypes, immune contexture and imaging modalities in relation to radiotherapy or chemoradiation outcomes; or (iv) described computational or multiomics models integrating molecular and clinicopathological features to predict treatment response. Purely preclinical experiments without direct clinical correlates were not included.

From eligible studies we extracted cohort characteristics, treatment modality (TMT, chemoradiation alone, NAC+RC), assay platform (e.g., immunohistochemistry, RNA sequencing, targeted panels), biomarker definitions, and reported performance metrics such as hazard ratios, odds ratios, AUCs, and calibration measures. Findings are presented as a narrative synthesis highlighting methodological strengths, limitations, and translational challenges, with an emphasis on decision-making for bladder preservation versus RC.

## 3. Endpoints and Patient Selection

### 3.1. Endpoint Specification and Harmonization for Computational Modeling

In computational prediction studies, endpoint specification is often a determinant of whether a model learns clinically meaningful signals. When cohorts are pooled, staging approaches, treatment delivery, and response assessment might not be uniform—imprecise endpoint definitions can introduce label noise and degrade transportability [3,5]. For this reason, prediction studies should pre-specify endpoints and describe their operational criteria with sufficient granularity to permit replication.

Complete response (CR) following induction/initial chemoradiation is typically defined at response assessment by absence of visible tumor with negative biopsies and/or cytology and is frequently treated as an early surrogate for subsequent bladder control in major bladder-preservation series [4,11]. By contrast, invasive intravesical recurrence (histologically confirmed ≥T2 recurrence) is a more unambiguous event from a patient perspective, as it generally precipitates consideration of salvage radical cystectomy (RC) and represents definitive failure of bladder preservation [5,8]. Bladder-intact survival, which integrates survival with preservation of the native bladder, is often more closely aligned with the therapeutic intent of organ-sparing management than overall survival alone and may be preferable when comparing strategies designed to preserve quality of life [4,5]. Salvage RC occupies a dual role in this literature—as both an outcome of interest and an intervention—and meta-analytic and prognostic syntheses provide estimates of its incidence, outcomes, and associated risk factors after bladder-sparing therapy [8,12].

### 3.2. Patient Selection Variables as Model Prerequisites

Before molecular features are introduced, risk models should incorporate clinicopathological determinants that influence both the probability of durable bladder preservation and the likelihood of being selected for TMT. Without these variables, apparent “biomarker effects” may reflect selection practices rather than biology, undermining interpretability and external validity. Guideline summaries and bladder-preservation reviews consistently emphasize selection based on local disease characteristics, feasibility of complete debulking, and histologic context [13,14].

Concomitant carcinoma in situ (CIS) is commonly interpreted as a marker of field-change biology and heightened intravesical recurrence propensity; accordingly, CIS status is frequently embedded in selection frameworks for bladder preservation [13,14]. Hydronephrosis is similarly treated as a surrogate for more advanced local disease and/or ureteral involvement and is repeatedly emphasized in guideline-based selection discussions [13,14]. The completeness of TURBT and overall debulking feasibility remain central: combined-modality series repeatedly associate maximal resection with response and bladder-intact outcomes, supporting explicit encoding of debulking status in computational datasets [4,11,14]. Histologic features and adverse pathological factors further refine risk. Guideline summaries further underscore the relevance of histology in treatment selection, knowing that different tumor variants and subtype biology can potentially display divergent sensitivity to chemoradiation across histologic contexts [13]. Where available, lymphovascular invasion should also be documented, given its independent association with survival and recurrence outcomes in cystectomy cohorts and its plausible implication as a marker of aggressive biology relevant to bladder-preservation failure [15].

## 4. DDR Genomics as Response Prediction Biology: *ERCC2* and Related Pathways

### 4.1. Biological Rationale

Ionizing radiation generates variable DNA lesions, among which double-strand breaks are often considered the most consequential for cytotoxicity, given the requirements for coordinated detection, checkpoint activation, and repair. The DNA damage response (DDR) orchestrates these processes across multiple repair pathways and cell-cycle control nodes. Therefore, variation in DDR capacity is plausibly linked to radiotherapy sensitivity [16]. Within trimodality therapy (TMT), concurrent chemotherapy is commonly deployed for radiosensitization, further increasing the burden of therapy-induced DNA damage. Under this framework, response can be conceptualized as a balance between damage induction (radiation ± chemotherapy) and the tumor’s ability to resolve that damage through repair mechanisms [16]. DDR alterations, accordingly, have been proposed as candidate predictive biomarkers for complete response, durable local control, and reduced probability of salvage cystectomy within bladder-preservation pathways [16].

A practical corollary is that DDR markers are more likely to function as predictive variables—modulating benefit from DNA-damaging therapy—than as generic prognostic features. Their clinical value, if confirmed, would therefore be expected to emerge most clearly when linked to treatment-specific endpoints such as CR after induction, invasive intravesical recurrence following chemoradiation, and the subsequent requirement for salvage cystectomy [16].

### 4.2. ERCC2 and Nucleotide Excision Repair

Among DDR candidates in urothelial carcinoma, *ERCC2* has received sustained attention because of its central role in nucleotide excision repair (NER) and its association with platinum susceptibility. In muscle-invasive urothelial carcinoma, somatic *ERCC2* mutations were reported to correlate with cisplatin sensitivity, positioning *ERCC2* as a candidate predictive biomarker for platinum responsiveness [17]. This observation is clinically salient for TMT, where cisplatin is frequently used as a radiosensitizer; thus, mechanisms that enhance platinum susceptibility may, by extension, increase the likelihood of effective chemoradiation in cisplatin-containing regimens [16,17].

In experimental studies, *ERCC2* helicase domain mutations have been shown to confer NER deficiency and to drive cisplatin sensitivity in muscle-invasive bladder cancer models, supporting causal inference beyond association alone [18]. Taken together, *ERCC2*/NER impairment constitutes a biomarker axis that can be incorporated into integrative prediction models for bladder preservation, ideally in combination with clinical selection variables and complementary transcriptomic state measures to improve calibration and external validity [16,17,18].

### 4.3. DDR Markers Beyond ERCC2: MRE11 and Radiotherapy Outcomes

Beyond NER, other DDR-linked markers have been evaluated in the context of radiotherapy response. MRE11 expression has been reported to predict cause-specific survival following radical radiotherapy for MIBC, suggesting that DDR pathway readouts may capture clinically relevant variation in radiation sensitivity [19]. More recently, MRE11 protein expression has been assessed in TURBT specimens from MIBC patients managed with TMT using an automated quantitative immunofluorescence (AQUA) approach, with a normalized MRE11 threshold used to stratify outcomes [20]. In that analysis, higher MRE11 expression was associated with reduced bladder cancer–specific mortality and improved overall survival, and the association persisted after adjustment for age, comorbidity, and additional risk factors, consistent with an effect that is not readily explained by baseline clinical differences alone [20]. While cut-point selection and assay harmonization remain non-trivial barriers to clinical implementation, these findings support continued evaluation of MRE11 as a candidate marker of heightened chemoradiation susceptibility [19,20].

### 4.4. DDR and Immunotherapy Responsiveness: Future Implications for Multi-Modal Strategies

DDR status may also influence immunotherapy responsiveness. Alterations in DDR and repair genes have been associated with clinical benefit from PD-1/PD-L1 blockade in advanced urothelial cancer, raising the possibility that DDR phenotypes help shape immune responsiveness and may be relevant to future combined-modality regimens [21]. This consideration is increasingly pertinent as bladder-preservation strategies are expected to incorporate immunotherapy peri-radiation or peri-operatively, and as trial designs move toward biomarker-informed escalation or de-escalation paradigms [22]. Within this emerging landscape, the principal translational challenge is to define which DDR features are predictive in the specific context of chemoradiation-based bladder preservation, rather than extrapolating uncritically from platinum-only or metastatic immunotherapy settings [16,21,22].

## 5. Transcriptomic and Immune Stratification of MIBC for TMT: Classification Frameworks, Quantification Methods, and Translational Constraints

### 5.1. Classification Frameworks: Basal–Luminal Lineage and Consensus Molecular Classes

Gene-expression profiling in muscle-invasive bladder cancer (MIBC) has repeatedly identified basal and luminal lineage patterns as dominant axes of transcriptional variation, establishing “subtype” as a composite representation of coordinated differentiation and pathway activity that is measurable in pre-treatment TURBT material [23]. The relevance to bladder preservation is supported by TMT-cohort analyses applying TURBT transcriptomics: in 189 MIBC cases classified as basal (45%), basal claudin-low (13%), infiltrated luminal (17%), and luminal (33%), infiltrated luminal tumors had a lower salvage cystectomy rate (12% vs. 34% in other subtypes; *p* = 0.08) and luminal tumors showed a non-significant trend toward improved overall survival (HR 0.63; *p* = 0.1), while complete response differences were not statistically significant [24].

Large-scale multi-omic efforts reinforce the view that transcriptomic classes are not merely labels but proxies for coordinated pathway activity and microenvironmental states. In TCGA (n = 412), 58 significantly mutated genes were reported; within the expression-based subgrouping, a high-mutation subset exhibited 75% five-year survival, while a “neuronal” expression subtype was associated with poor survival despite limited overt neuroendocrine histology [25]. Against the backdrop of historical inter-study discordance in subtype nomenclature and classifier behavior, the consensus molecular classification sought to improve interoperability by integrating 1750 transcriptomes across 16 published datasets and two additional cohorts; six harmonized classes were defined with reported frequencies (luminal papillary 24%, luminal non-specified 8%, luminal unstable 15%, stroma-rich 15%, basal/squamous 35%, neuroendocrine-like 3%), alongside a single-sample classifier intended to support reproducible assignment [26]. More broadly, the centrality of transcription-derived taxonomy is consonant with pan-cancer evidence that cell-of-origin patterns can dominate molecular classification across tumor types [27].

### 5.2. Immune Contexture: Prognostic Relevance and Computational Deconvolution

Immune contexture constitutes a further stratification dimension with potential relevance to TMT-specific endpoints, particularly where tumor differentiation and microenvironmental composition vary in parallel. Associations between immune contexture, differentiation features, and clinical outcomes have been reported in bladder cancer, providing a rationale for joint modeling rather than treating immune infiltration as an unmeasured background variable [28]. Complementary TCGA-based analyses have identified immune-related genes associated with prognosis and risk, yielding candidate immune signatures that may be prioritized for evaluation in bladder-preservation cohorts using endpoints such as CR, invasive recurrence, bladder-intact survival, and salvage cystectomy [29]. From an operational standpoint, immune composition can be estimated from bulk transcriptomes using standardized deconvolution approaches (e.g., CIBERSORT), enabling comparable immune infiltration features that can be integrated with subtype calls and pathway scores in multivariable models [30].

### 5.3. Reproducible Transcriptomic Feature Engineering: Pathway Scoring and Discovery Platforms

Methods that reduce platform dependence facilitate cross-cohort comparability and model transportability. GSVA provides pathway-level scoring of biological programs as continuous model-ready variables suitable for integration with subtype labels and immune deconvolution outputs [31]. Limma supports reproducible differential expression workflows for RNA-seq and microarrays and remains a common foundation for signature derivation and validation in translational cohorts [32]. Single-cell RNA sequencing provides a framework to resolve immune heterogeneity and tumor–immune interactions, supporting discovery and refinement of immune programs that can later be approximated in bulk cohorts [33].

### 5.4. Translational Constraints: Endpoint Heterogeneity, Assay Variability, and Clinical Interpretability

Subtype and immune biomarkers should be interpreted considering endpoint and assay heterogeneity. Subtype-stratified neoadjuvant chemotherapy literature highlights potential label noise from TURBT-associated downstaging (≈10% pCR without neoadjuvant therapy; 38% of pathologic responses attributed to TURBT), cohort-size limitations (16–251; median 85), heterogeneity in subtype granularity (3–7), and inconsistent response definitions, all of which are directly relevant to TMT, where CR assessment and salvage cystectomy thresholds vary across programs [34]. Consistent with focused commentary in the TMT setting, integration of molecular subtyping with tumor infiltration measures remains incompletely resolved; therefore, subtype-based pre-TMT stratification is best positioned as hypothesis-generating and trial-enabling, pending standardized, externally validated associations with CR, invasive recurrence, and salvage cystectomy endpoints [26,35].

## 6. Radiogenomics and Digital Pathology as Noninvasive Surrogates

### 6.1. Radiomics and Radiogenomics for Bladder Cancer Outcomes

Radiomics and radiogenomics attempt to distinguish quantitative phenotypes from routine imaging and relate these features to oncologic outcomes and, in radiogenomics, to underlying molecular programs. The current review literature conveys guarded optimism: potential clinical utility is repeatedly noted, yet substantial methodological heterogeneity—spanning acquisition protocols, segmentation practices, feature engineering, and validation strategy—continues to limit comparability across studies and reinforces the need for standardization and rigorous external validation prior to clinical deployment [36,37].

Although much of the bladder radiomics experience has been anchored in CT, MRI radiomics is increasingly explored as a complementary modality. In a JCO 2025 abstract in MIBC treated with neoadjuvant pembrolizumab followed by cystectomy (PURE-01 context), models derived from post-treatment MRI radiomic features outperformed pre-treatment models for predicting pCR (test AUC ≈ 0.83 vs. ≈0.66) and achieved high performance for major response (test AUC ≈ 0.92). These data suggest that mpMRI radiomics can capture treatment-modified tumor state and may, with appropriate endpoint-specific validation, serve as a noninvasive response surrogate in bladder-preservation pathways [38]. This cohort reflects neoadjuvant immunotherapy followed by cystectomy and is not a TMT dataset; its value here is conceptual (treatment-modified imaging phenotypes) rather than evidence of TMT prediction performance.

### 6.2. Deep Learning and Response Assessment on CT

Imaging is also embedded in routine response assessment and surveillance, and radiomics offers a means of formalizing what is otherwise a predominantly qualitative interpretation. CT-based radiomics with deep learning has been evaluated for bladder cancer treatment response assessment, supporting feasibility of deriving discriminative imaging features for response classification [39]. For TMT, the potential utility is principally adjunctive: imaging-derived signatures could complement cystoscopic and biopsy-based assessment by providing an additional standardized signal of residual disease or early progression risk, contingent on validation against TMT-relevant outcomes such as invasive intravesical recurrence and the requirement for salvage cystectomy [39].

### 6.3. Digital Pathology and AI in Bladder Cancer

Whole-slide imaging (WSI) and computational pathology offer a parallel, tissue-based—yet scalable—approach to quantifying tumor morphology and microenvironmental composition. A dedicated review on artificial intelligence in bladder cancer summarizes the emerging landscape of WSI-based modeling and its potential to infer clinically relevant phenotypes, including grade- and invasion-related features and signals linked to immune infiltration [40]. In bladder-preservation workflows, these approaches are methodologically appealing because WSI features can be extracted from diagnostic TURBT specimens and may provide surrogates for transcriptomic programs (e.g., basal/squamous differentiation, stromal enrichment, immune contexture) that would otherwise require RNA profiling [40].

### 6.4. Integration with Multi-Omics Models: Translational Considerations

Radiogenomics and computational pathology may be particularly valuable when comprehensive sequencing is not feasible, and they can augment multi-omics prediction by contributing orthogonal phenotypes that may improve interpretability and mitigate overfitting in high-dimensional models [36,40]. The longitudinal signal implied by MRI radiomics—where post-treatment features appear more predictive than baseline features—also supports modeling strategies that incorporate on-treatment imaging to refine early prediction of response and failure [38]. At the same time, the translational barriers remain familiar: imaging standardization, segmentation reproducibility, calibration, and independent external validation across scanners, institutions, and treatment regimens are repeatedly emphasized as prerequisites for decision support in contemporary reviews [36,37].

## 7. When to Recommend Cystectomy: Upfront Versus Salvage (Evidence and Guideline Framing)

European guidelines emphasize that eligibility for bladder preservation should be determined by clinical and pathological selection factors (including CIS status, hydronephrosis, completeness of TURBT/debulking, and histology), which should structure counseling toward TMT versus upfront radical cystectomy (RC) [13]. Systematic reviews similarly indicate that TMT outcomes are strongly selection-dependent, supporting upfront RC discussion when adverse features cluster and the probability of durable bladder-intact control is low [3,6].

Salvage RC is an expected component of bladder-preservation pathways and is contextualized by long-term pooled outcomes from RTOG combined-modality protocols [5]. Meta-analytic evidence quantifies the incidence and oncologic outcomes of salvage cystectomy after bladder-sparing therapy, supporting pre-treatment counseling that salvage surgery is anticipated in a subset of patients and can remain oncologically effective; prognostic syntheses further support stratification for salvage RC risk after TMT [8,12]. In a retrospective single-centre comparison (n = 265), salvage cystectomy after TMT was associated with higher late complication rates (any late complications: HR 2.3, *p* = 0.02; major late complications: HR 2.1, *p* < 0.05) without differences in intra-operative or early complications, disease-specific survival (*p* = 0.8), or overall survival (*p* = 0.9) compared with primary cystectomy groups [41].

In practice, upfront RC should be favored when guideline-defined adverse selection factors and individualized risk estimates exceed the patient’s acceptable threshold, whereas TMT could be offered when maximal debulking is feasible and predicted probabilities of complete response and bladder-intact survival are favorable under intensive surveillance [4,5,7,13]. Salvage RC should be recommended promptly in cases of non-response or invasive intravesical recurrence, consistent with its established role as a potentially curative intervention after TMT failure [5,8].

## 8. Precision Framework for TMT Selection and Cystectomy Timing in MIBC

### 8.1. Domains of Interest and Integration for Treatment Selection

A precision-oriented selection framework for MIBC can be formulated as a decision matrix integrating (i) tumor biology, (ii) clinicopathological risk, and (iii) patient-level constraints to guide selection of trimodality therapy (TMT) versus upfront radical cystectomy (RC) and to define thresholds for salvage cystectomy [3,13]. The biological domain is structured around transcriptomic class membership, immune contexture, and DNA damage response (DDR) phenotypes, which can be quantified from TURBT tissue using validated classifiers, immune deconvolution/pathway scoring, and targeted DDR assays [16,17,18,19,26,28,30,31]. Clinicopathological selection determinants (e.g., CIS, hydronephrosis, completeness of debulking, histology) remain required for confounding control and interpretability, while patient factors determine feasibility and acceptable risk thresholds [7,13,14]. These factors and their influence are further elaborated on in Table 1.

**Figure 1:**
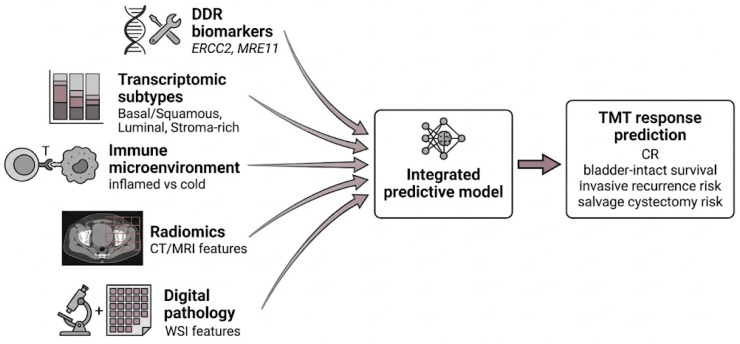
Multi-omics determinants of TMT response. (**DDR**: DNA damage response; **TMT**: trimodality therapy; **CR**: complete response; **WSI**: whole-slide imaging; **CT**: computed tomography; **MRI**: magnetic resonance imaging.)

A staged decision-support workflow can therefore be conceptualized as: (i) standardized baseline staging and maximal TURBT with multidisciplinary review; (ii) TURBT-based molecular profiling to derive subtype, immune, and DDR features; (iii) interpretable multimodal integration with the clinical backbone to generate calibrated risk estimates; and (iv) mapping risk to recommendation tiers with dynamic reassessment after induction chemoradiation using cystoscopic and radiologic response, and potentially liquid-biopsy signals in future adaptive strategies [4,5,13,26,30,31,42,43,44]. This framework, illustrated in Figure 1, should be positioned as hypothesis-generating and trial-enabling, pending derivation and independent validation in well-annotated TMT cohorts [3,6,8].

**Table 1:** Key biomarkers involved in the precision-oriented decision matrix for trimodality therapy (TMT) selection and cystectomy timing in muscle-invasive bladder cancer (MIBC) (conceptual).

Factor (Domain + Variable)	Pre-TMT Operationalization	Decision Relevance
**Clinicopathologic: CIS status**	TURBT pathology; document concomitant CIS [13,14].	**May favor TMT**: CIS absent. **May favor upfront RC/lower salvage threshold:** CIS present given higher intravesical failure risk and reduced suitability for bladder preservation [3,13].
**Clinicopathologic: hydronephrosis**	Baseline imaging; record hydronephrosis [13,14].	**May favor TMT:** no hydronephrosis. **May favor upfront RC/lower salvage threshold:** hydronephrosis as a marker of adverse local disease [13].
**Clinicopathologic: TURBT completeness/debulking feasibility**	Operative report + pathology; document maximal TURBT [13,14].	**May favor TMT:** maximal debulking feasible/achieved. **May favor upfront RC/lower salvage threshold:** incomplete debulking or inability to achieve maximal TURBT (reduced probability of durable bladder control) [4,5].
**Clinicopathologic: histology/variants**	Standard histopathology; record variant features [13].	**May favor TMT:** histology compatible with bladder preservation in guideline-based selection. **May favor upfront RC:** adverse histologic contexts where bladder preservation suitability is reduced [13].
**Pathology risk: lymphovascular invasion (if available)**	TURBT pathology when reported (limited sensitivity) [15].	**May favor TMT:** LVI absent (lower-risk biology). **May favor upfront RC/lower salvage threshold:** LVI present as aggressive-biology marker associated with worse recurrence/survival in cystectomy cohorts [15].
**Transcriptome: consensus molecular subtype**	RNA from TURBT; assign consensus class with validated classifier [26].	**May favor TMT:** subtypes associated with more favorable biology for local control (to be validated against TMT endpoints). **May favor upfront RC/lower salvage threshold**: subtypes associated with aggressive programs (requires endpoint-specific validation) [26,35].
**Immune: immune contexture**	Bulk RNA; immune deconvolution and immune program scoring [28,30].	**May favor TMT**: immune-inflamed contexture consistent with improved outcomes in bladder cancer. **May favor upfront RC/lower salvage threshold:** immune-cold/excluded phenotypes (requires validation in TMT cohorts) [28,30].
**Immune: immune-risk signatures**	Candidate immune genes/signatures prioritized from TCGA-type analyses for testing [29].	**Favors TMT vs. RC**: directionality must be defined by TMT-specific validation; intended for risk stratification rather than deterministic selection [29,35].
**DDR: MRE11**	TURBT immunohistochemistry; quantify expression [19,20].	**May favor TMT:** DDR marker profile associated with radiotherapy outcomes. **May favor upfront RC/lower salvage threshold:** DDR profile associated with poorer radiotherapy-related outcomes [19,20].
**DDR: *ERCC2*/NER alterations**	Targeted DNA sequencing from TURBT where feasible [17,18].	**May favor TMT:***ERCC2*/NER alterations associated with cisplatin sensitivity and NER deficiency, potentially increasing benefit from platinum-based radiosensitization. **May favor upfront RC/lower salvage threshold:** absence of sensitizing DDR signals does not preclude TMT but reduces biologic rationale for enhanced chemo-radiation sensitivity [14,17,18].
**Feature engineering: pathway program scores**	GSVA-based continuous program scoring from bulk expression [31].	**May favor TMT vs. RC:** program directionality must be defined by TMT-specific model calibration; pathway scores provide continuous features enabling risk-tier assignment rather than binary eligibility [31].
**Imaging: CT radiomics for response assessment**	Standardized CT acquisition/segmentation; extract radiomic/deep-learning features [39].	**May favor TMT:** favorable early imaging response signature may support continuation. **May favor early salvage/lower salvage threshold:** adverse imaging might suggest residual disease risk [39].
**Imaging: MRI radiomics (treatment-modified state)**	mpMRI radiomics; compare baseline vs. on-treatment/post-treatment features [38].	**May favor continued organ-preservation strategy:** favorable post-treatment MRI radiomics associated with response (pCR AUC = 0.83 post vs. = 0.66 pre; major response AUC = 0.92). **May favor early salvage strategy:** adverse post-treatment imaging [38].
**Digital pathology: whole-slide imaging (WSI)/AI features**	WSI from TURBT; derives morphologic and microenvironmental features [40].	**May favor TMT:** WSI features consistent with favorable differentiation/immune contexture. **May favor upfront RC/lower salvage threshold:** WSI features consistent with aggressive invasion patterns or adverse microenvironmental signals [40].
**Modeling: interpretable multimodal integration**	Late fusion + attribution frameworks integrating clinical + molecular ± imaging features [43].	**May favor TMT vs. RC:** enables calibrated individualized risk tiers (e.g., predicted CR, invasive recurrence, salvage RC risk) rather than deterministic single-marker selection [43].

**DDR**: DNA damage response; **CIS**: carcinoma in situ; **TURBT**: transurethral resection of bladder tumor; **TMT**: trimodality therapy; **RC**: radical cystectomy; **LVI**: lymphovascular invasion; **NER**: nucleotide excision repair; **GSVA**: gene set variation analysis; **CT**: computed tomography; **mpMRI**: multiparametric magnetic resonance imaging; **pCR**: pathological complete response; **AUC**: area under the curve; **WSI**: whole-slide imaging; **AI**: artificial intelligence; **CR**: complete response.

**Figure 2:**
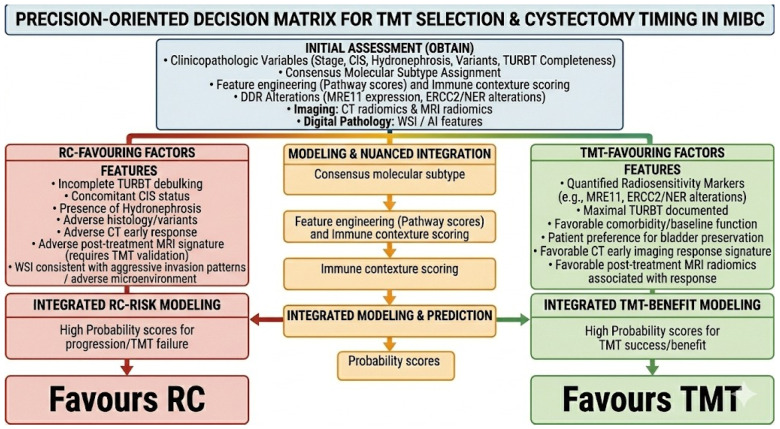
Precision-Oriented Decision Matrix for TMT Selection & Cystectomy Timing in MIBC. (**MIBC**: muscle-invasive bladder cancer; **TMT**: trimodality therapy; **RC**: radical cystectomy; **CIS**: carcinoma in situ; **TURBT**: transurethral resection of bladder tumor; **DDR**: DNA damage response; **NER**: nucleotide excision repair; **WSI**: whole-slide imaging; **AI**: artificial intelligence; **CT**: computed tomography; **MRI**: magnetic resonance imaging; **pCR**: pathological complete response; **AUC**: area under the curve.)

Figure 2 illustrates a precision-oriented decision matrix for TMT selection and cystectomy timing in MIBC. The framework begins with an initial assessment (obtain) to gather comprehensive patient and tumor data, including clinicopathologic variables, consensus molecular subtype assignment, and DDR alterations. Additionally, this initial phase integrates imaging (CT and MRI radiomics) and digital pathology (WSI/AI features). Specific features are evaluated to guide the optimal treatment strategy, categorized as either RC-favouring factors or TMT-favouring factors. The novelty of this framework lies in its explicit integration of clinicopathologic selection variables with multi-omics and imaging-derived features into a unified directional scoring system, rather than treating these domains independently.

### 8.2. Toward a Quantitative Scoring Framework for TMT Selection

The precision framework outlined in Figure 2 synthesizes multiple decision domains in a qualitative manner. To operationalize this framework for clinical decision support and prospective trial design, we propose a hypothesis-generating, weighted scoring approach in which clinicopathologic and omics features are assigned directional scores that sum toward a TMT-favoring or RC-favoring recommendation (Table 2). This approach is explicitly conceptual and should be regarded as a scaffold for future prospective derivation and validation, not as a validated clinical tool. In this schema, TMT-favoring factors receive positive scores and RC-favoring factors receive negative scores; the summed weighted score provides a composite signal for treatment direction, which can be used to support shared decision-making discussions.

Critically, factors should not be weighted equally. Clinicopathologic variables—completeness of TURBT, presence of hydronephrosis—carry the most consistent and mature evidence across multi-institutional series and guideline documents, and are therefore assigned higher weights. Molecular biomarkers (DNA damage response [DDR] alterations, transcriptomic subtype, MRE11 expression) carry meaningful but less independently externally validated evidence in the TMT-specific context and are assigned proportionally lower weights. These weights are hypothesis-generating; final weights should be derived empirically from multivariable modeling in prospectively annotated TMT cohorts.

## 9. From Code to Clinic

### 9.1. Data Requirements and Public Resources

Moving computational genomics from exploratory analyses into decision support for TMT will likely depend on datasets that are not merely large, but also harmonized: treatment details (including radiosensitizer selection), standardized endpoint definitions (CR, invasive recurrence, bladder-intact survival, salvage cystectomy), and synchronized multimodal predictors spanning molecular assays, imaging, and pathology need to co-exist within the same analytic frame [3,4,5]. TCGA remains an indispensable molecular reference for MIBC biology, yet it was not designed as a TMT registry and does not systematically encode bladder-preservation workflows, response assessments, or salvage cystectomy pathways [25]. In practice, this leaves multicenter TMT cohorts with consistent annotation as the more plausible substrate for model derivation and genuinely independent external validation [8,12].

### 9.2. Framework Validation and Implementation

The scoring framework should be embedded as a prespecified stratification variable in future TMT-enriched trials, including SWOG/NRG S1806 and ECOG-ACRIN EA8185 (INSPIRE). All component variables should be prospectively collected at enrollment, and the score should be validated against prespecified endpoints (complete response, invasive intravesical recurrence, bladder-intact survival, salvage cystectomy) using calibration curves and decision-curve analysis to define clinically meaningful thresholds. Once externally validated and calibrated, actionable score tiers could be defined—for example, a highly positive composite score favoring TMT, a strongly negative score favoring upfront RC discussion, and intermediate scores triggering multidisciplinary team review. This structure maps onto and extends existing European Association of Urology (EAU) guideline selection criteria, which already incorporate multiple clinical selection factors in a semi-quantitative manner, and would provide a transparent, reproducible, and patient-communicable tool for shared decision-making. The primary goal of such a scoring system is not to replace clinical judgment but to structure the integration of heterogeneous evidence domains into a consistent, communicable risk estimate that can be refined prospectively and, ultimately, embedded into future clinical practice guidelines.

### 9.3. Methodological and Implementation Limitations

Experience with bladder cancer risk modeling in adjacent clinical contexts suggests recurrent vulnerabilities—small-cohort overfitting, limited interpretability, and a shortfall of robust external validation—which are likely to recur unless addressed explicitly [45]. Within TMT specifically, endpoint heterogeneity and institutional variation in selection and salvage thresholds may distort apparent biomarker associations if not modeled as part of the data-generating process, a concern emphasized in comparative syntheses and systematic reviews [3,6]. Imaging-based models face additional obstacles: variability in acquisition and segmentation, coupled with inconsistent validation practices, continues to constrain translation, as highlighted in contemporary radiomics/radiogenomics reviews [36,37]. Despite growing multi-omics evidence, prospective validation of integrated biomarkers in trimodality therapy cohorts remains limited, representing one of the main barriers to clinical implementation.

## 10. Conclusions

Avoiding preventable TMT failures requires a unified framework in which clinical selection (CIS, hydronephrosis, maximal TURBT feasibility, and histology) is combined with mechanistic biomarkers (DDR/*ERCC2* and related pathways; MRE11), transcriptomic state (consensus subtypes, hypoxia/EMT/stroma programs), and immune contexture quantified by reproducible computational methods [13,17,19,26,30]. Radiogenomics and digital pathology offer scalable, noninvasive surrogates that can extend prediction to settings with limited sequencing and can strengthen multi-modal models when integrated thoughtfully [36,39,40]. The clinical goal is not simply higher AUC, but calibrated, externally validated decision support that defines thresholds for recommending upfront RC versus bladder preservation with readiness for timely salvage RC, reflecting the evidence base and guideline selection principles for MIBC [3,7,8,13].

## 11. Future Directions

Two lines of development appear poised to sharpen the practical boundary between “bladder preservation with confidence” and “early conversion to cystectomy.”

Regarding local therapy omission for MIBC, accumulating evidence from the RETAIN program and allied trials illustrates why this strategy remains firmly investigational and should not be adopted outside prospective trial settings. RETAIN-1, which enrolled DDR-mutated (*ATM*, *ERCC2*, *FANCC*, *RB1*) cT2–T3 MIBC patients achieving cCR to accelerated MVAC, failed to meet its primary endpoint of 2-year metastasis-free survival (72.9%), with 40% of per-protocol surveillance patients developing NMIBC recurrence and only 17% remaining alive, metastasis-free, and bladder-intact at primary analysis [46]. Although RETAIN-2 (adding nivolumab) met its primary endpoint (2-year MFS 77.4%), approximately 30% of active surveillance patients with recurrent NMIBC in that cohort went on to develop metastatic disease, and the integrated ctDNA analysis revealed an NPV of only 32% for local-only recurrence—underscoring that even in biomarker-selected patients, ctDNA-negative status cannot safely exclude intravesical residual disease [46,47]. The IBCG-GSRGT consensus (2025) explicitly states that omission of local definitive therapy “should not guide routine clinical decision-making” and must remain confined to clinical trials [48]. Rather than pursuing the omission of local treatment altogether, the more productive and immediately actionable imperative is to improve patient selection for trimodality therapy itself—refining who is most likely to achieve durable bladder preservation with an intact organ through better clinical staging, more precise molecular stratification, and prospectively validated decision frameworks.

As an adjunct for treatment choice alongside plasma ctDNA, urinary tumor DNA (utDNA) has emerged as a complementary liquid biopsy modality with particular relevance to bladder cancer. Whereas ctDNA enters the bloodstream through apoptosis and tumor cell lysis, reflecting systemic tumor burden, utDNA fragments are shed directly from urothelial tumor cells into the urine, generating a high-concentration local signal from the intravesical compartment—the precise anatomic location where ctDNA performs most poorly. The critical nature of this gap was demonstrated in the integrated RETAIN-1/2 ctDNA analysis: ctDNA positivity had a PPV of 90% for residual disease at cystectomy, yet the NPV for local-only recurrence was only 32% [46]. This means that ctDNA-negative status does not reliably exclude intravesical residual disease—the scenario most relevant to TMT surveillance and response-adapted omission strategies. A recent systematic review confirmed that utDNA demonstrates superior tumor detection capabilities for minimal residual disease, provides grade and staging information, and holds potential for supporting bladder preservation strategies [49]. As a surveillance tool for NMIBC recurrence, there is a reported sensitivity of 91% and specificity of 85% for high-grade recurrence [50]. These advances suggest that the future of local treatment omission and post-TMT surveillance and response-adapted strategies should integrate both compartments: ctDNA for systemic disease risk stratification and utDNA for intravesical residual disease detection [51].

Convergent, TMT-specific data further indicate that plasma ctDNA positivity may anticipate metastatic progression earlier than conventional assessment, supporting ctDNA-guided post-TMT surveillance as a plausible adjunct to existing cystoscopic and radiologic workflows rather than a replacement for them [52]. In parallel, advances in computational pathology are beginning to erode a long-standing operational constraint—namely, dependence on RNA-based assays for subtype assignment—by showing that deep-learning models applied to whole-slide images can predict molecular subtypes and clinical outcomes in MIBC, a direction that may enable broader, pre-treatment deployment of subtype-informed stratification in routine practice settings [53].

A second, closely related priority is to embed these biologic and computational signals into prospective trial architectures where endpoints and salvage pathways are prespecified and auditable. The addition of immunotherapy to bladder-preserving chemoradiotherapy is being tested in randomized or prospective frameworks, exemplified by SWOG/NRG S1806 evaluating concurrent chemoradiotherapy with or without atezolizumab in localized MIBC, and ECOG-ACRIN/NRG EA8185 (INSPIRE) testing chemoradiation with durvalumab in clinically node-positive disease [54,55]. Within such trials, it becomes feasible to define the evidentiary threshold at which a multimodal risk signature (subtype/immune/DDR, imaging, WSI features) should trigger intensified monitoring or earlier salvage cystectomy, while ctDNA kinetics can be evaluated as an adaptive signal for systemic progression risk in the post-TMT interval [46,52,54,55].

In future clinical practice, an initial decision between bladder-preserving trimodal therapy and upfront radical cystectomy might go beyond established clinicopathologic factors and will possibly take into consideration emerging molecular predictors, transcriptomic subtype classification, and immune contexture. Multimodal prediction frameworks integrating these domains may enable individualized estimation of complete response probability and invasive recurrence risk, thereby supporting precision-based treatment selection and timely consideration of salvage cystectomy.

## Figures and Tables

**Table 2:** Proposed Weighted Scoring System for TMT vs. Radical Cystectomy Selection in MIBC.

Factor	Favoring Direction	Proposed Score
Macroscopically complete TURBT (R0 debulking)	TMT	+2
Absence of hydronephrosis	TMT	+1
Absence of concomitant CIS	TMT	+1
Favorable DDR alteration (*ERCC2*, *ATM*, *FANCC*, or *RB1*)	TMT	+2
High MRE11 expression (AQUA quantification)	TMT	+1
Luminal or infiltrated luminal molecular subtype	TMT	+1
Favorable Post-Treatment MRI	TMT	+1
Concomitant CIS	RC	−1
Hydronephrosis	RC	−2
Incomplete or technically unfeasible TURBT	RC	−2
Basal/squamous or neuroendocrine-like molecular subtype	RC	−2
Adverse variant histology	RC	−2
Adverse WSI/AI patterns	RC	−1

Score interpretation is conceptual and hypothesis-generating. Summed scores are not validated clinical thresholds. Positive totals favor TMT consideration; negative totals favor upfront RC discussion; intermediate totals should trigger multidisciplinary team review. **MIBC**: muscle-invasive bladder cancer; **TMT**: trimodality therapy; **RC**: radical cystectomy; **TURBT**: transurethral resection of bladder tumor; **CIS**: carcinoma in situ; **DDR**: DNA damage response; **AQUA**: automated quantitative immunofluorescence; **WSI**: whole-slide imaging; **AI**: artificial intelligence; **MRI**: magnetic resonance imaging; **pCR**: pathological complete response; **CR**: complete response; **OS**: overall survival.

## Data Availability

No new data were created or analyzed in this study. Data sharing is not applicable to this article. All analyses were based on previously published datasets and clinical trial results available in the public domain and referenced throughout the text.

## Abbreviations

The following abbreviations are used in this manuscript:

AI	Artificial Intelligence
AQUA	Automated Quantitative Immunofluorescence
AUC	Area Under the Curve
cCR	Clinical Complete Response
CIBERSORT	Cell-Type Identification by Estimating Relative Subsets of RNA Transcripts
CIS	Carcinoma In Situ
CR	Complete Response
CT	Computed Tomography
ctDNA	Circulating Tumor DNA
DDR	DNA Damage Response
EAU	European Association of Urology
ECOG-ACRIN	Eastern Cooperative Oncology Group—American College of Radiology Imaging Network
EMT	Epithelial–Mesenchymal Transition
GSVA	Gene Set Variation Analysis
HR	Hazard Ratio
LVI	Lymphovascular Invasion
MFS	Metastasis-Free Survival
MIBC	Muscle-Invasive Bladder Cancer
mpMRI	Multiparametric Magnetic Resonance Imaging
MRI	Magnetic Resonance Imaging
MVAC	Methotrexate, Vinblastine, Doxorubicin, and Cisplatin
NER	Nucleotide Excision Repair
NMIBC	Non-Muscle-Invasive Bladder Cancer
NPV	Negative Predictive Value
NRG	NRG Oncology
OS	Overall Survival
pCR	Pathological Complete Response
PD-L1	Programmed Death-Ligand 1
PPV	Positive Predictive Value
RC	Radical Cystectomy
RNA	Ribonucleic Acid
RTOG	Radiation Therapy Oncology Group
SWOG	Southwest Oncology Group
TCGA	The Cancer Genome Atlas
TMB	Tumor Mutational Burden
TMT	Trimodality Therapy
TURBT	Transurethral Resection of Bladder Tumor
utDNA	Urinary Tumor DNA
VI-RADS	Vesical Imaging-Reporting and Data System
WSI	Whole-Slide Imaging
